# Serum Zn/Cu Ratio Is Associated with Renal Function, Glycemic Control, and Metabolic Parameters in Japanese Patients with and without Type 2 Diabetes: A Cross-sectional Study

**DOI:** 10.3389/fendo.2016.00147

**Published:** 2016-11-15

**Authors:** Hidetaka Hamasaki, Yu Kawashima, Hidekatsu Yanai

**Affiliations:** ^1^Department of Internal Medicine, National Center for Global Health and Medicine, Kohnodai Hospital, Chiba, Japan; ^2^Department of Radiology, Chiba University Hospital, Chiba, Japan

**Keywords:** type 2 diabetes, zinc, copper, eGFR, B-type natriuretic peptide

## Abstract

**Background:**

Zinc (Zn) and copper (Cu) may play a pivotal role in the pathogenesis of diabetes and diabetic complications by mediating oxidative stress. Both Zn deficiency and excess of Cu are associated with an increased risk of type 2 diabetes and cardiovascular disease. We aimed to investigate the relationships between serum Zn/Cu ratio and glycemic status, renal function, and metabolic parameters in patients with and without type 2 diabetes.

**Methods:**

We conducted a cross-sectional study on 355 subjects (149 type 2 diabetic and 206 non-diabetic) in whom serum Zn and Cu levels were measured at the same time. Associations between serum Zn/Cu ratio and clinical data were evaluated using multiple regression analysis. We also evaluated associations between serum Zn/Cu ratio and the prevalence of type 2 diabetes and glycemic control by multivariate logistic regression analysis.

**Results:**

Serum Zn/Cu ratio was positively associated with estimated glomerular filtration rate after adjustment for body mass index (BMI) (β = 0.137, *p* = 0.014). Plasma B-type natriuretic peptide levels were negatively associated with serum Zn/Cu ratio after adjustment for age, sex, and BMI (β = −0.258, *p* = 0.032). In patients with type 2 diabetes, serum Zn/Cu ratio was negatively associated with plasma HbA1c levels after adjustment for age, sex, and BMI (β = −0.239, *p* = 0.003). In addition, multivariate logistic regression analysis revealed that the highest quartile of serum Zn/Cu ratio was associated with a reduced risk of poor (HbA1c ≥ 7%) glycemic control (odds ratio = 0.382; 95% confidence interval, 0.165–0.884; *p* = 0.025) in patients with type 2 diabetes.

**Conclusion:**

Serum Zn/Cu ratio was favorably associated with renal function in all subjects and glycemic control in patients with type 2 diabetes. The Zn/Cu ratio, in addition to the individual serum levels of trace elements, is important for metabolism in humans.

## Introduction

Diabetes is a metabolic disease characterized by defects in insulin secretion, insulin sensitivity, or both. Trace elements, such as zinc (Zn) and copper (Cu), may play a pivotal role in the pathogenesis of diabetes and diabetic vascular complications by mediating oxidative stress (1–4). Several studies have shown that both Zn deficiency and excess of Cu are associated with an increased risk of type 2 diabetes and cardiovascular disease (CVD) (2, 3, 5–9).

Zn is a critical trace element in human health. Zn has a potential to be utilized for the treatment of type 2 diabetes; however, the epidemiologic evidence suggests that the effect of Zn on type 2 diabetes remains unclear (10). Up to 85% of the whole body Zn content is found in muscle and bones, with 11% in the skin and liver (11). Zn is an indispensable cofactor for more than 300 enzymes involved in metabolism and also reportedly plays a role in aging, immune system, apoptosis, and oxidative stress (11). Although the effect of zinc supplementation in the improvement of oxidative stress is controversial, one of the causes that the oxidative stress is present in patients with type 2 diabetes is the change in zinc metabolism (4). Moreover, a number of studies have suggested that matrix metalloproteinases which include a Zn ion-binding site are associated with the progression of diabetic microvascular complications and diabetic tendon disorders (12). Recent studies have demonstrated that the islet-restricted zinc transporter, ZnT8 (*SLC30A8*), regulates insulin secretion (13) and hepatic insulin clearance (14), suggesting that Zn is a key biological factor in glucose homeostasis and the risk of developing type 2 diabetes (15).

Cu has an integral role in many enzymatic activities involved in modifying oxidative stress. Free Cu ions have catalytic activity in the generation of highly reactive hydroxyl radicals (16). Disruption of Cu homeostasis induces oxidative damage by free radicals; such Cu toxicity is associated with disrupted lipid metabolism, hepatic disorders, neurodegenerative disorders, and atherogenesis (17, 18). Cu ion may also play a protective role in the accumulation of human islet amyloid peptide, which is the major component of amyloid deposits in pancreatic β-cells of type 2 diabetic patients; however, whether or not Cu have a protective role in the etiology of type 2 diabetes is not clarified (19, 20). Excess of Cu under inflammatory conditions trigger oxidative stress which are present in chronic diseases (21). On the other hand, increased Zn ion levels may provide a protective effect against Cu toxicity by competing for Cu binding sites (16).

Epidemiological and biological studies have indicated that an imbalance between serum Zn and Cu levels is a causative factor for various diseases, particularly diabetes and CVD (2, 3, 5–9). However, there is a lack of evidence regarding the association between serum Zn/Cu ratio and metabolic parameters in humans. In this study, we aimed to investigate the relationships between serum Zn/Cu ratio and various parameters, such as hematological parameters, glycemic status, lipid profile, renal function, and body composition, in patients with and without type 2 diabetes.

## Materials and Methods

### Subjects

Between April 2010 and November 2014, a total of 355 individuals (149 type 2 diabetic patients and 206 non-diabetic patients) who measured both serum Zn and Cu at our hospital were investigated retrospectively. Exclusion criteria were type 1 diabetes, malnutrition [serum albumin (Alb) < 3.0 g/dl], and anemia [plasma hemoglobin (Hb) levels < 10 g/dl]. Chronic kidney disease was defined as an estimated glomerular filtration rate (eGFR) less than 60 mL/min/1.73 m^2^. Patients were diagnosed as having type 2 diabetes according to the Japanese diagnostic criteria for type 2 diabetes (22). Briefly, diabetic type was diagnosed if subjects met the following criteria: fasting plasma glucose (PG) level of ≥126 mg/dl or casual PG level of ≥200 mg/dl or HbA1c level ≥6.5%. Patients with type 1 diabetes were excluded. If such conditions were confirmed more than once in the past or the presence of typical symptoms of diabetes and definite diabetic retinopathy were detected, patients were diagnosed as having type 2 diabetes. However, HbA1c level ≥6.5% alone cannot be defined as diabetes (22).

The patients were anonymized to protect their personal information. The study protocol was approved by the Medical Ethics Committee of the National Center for Global Health and Medicine Kohnodai Hospital (Reference No. NCGM-G-001912), and the study was performed in accordance with the Declaration of Helsinki.

### Anthropometric Measurement

Height and weight were measured using a rigid stadiometer and calibrated scales (seca 764, seca Co., Ltd., Birmingham, UK). Body mass index (BMI) was calculated as body weight in kilograms divided by the square of body height in meters.

### Blood Examination

We measured blood cell count, Alb, blood urea nitrogen, creatinine, eGFR, Zn, and Cu. Serum Zn and Cu levels were measured by atomic absorption spectrophotometry (Z6100, Hitachi Power Solutions Co., Ltd., Ibaraki, Japan) (23, 24). eGFR was calculated using the revised equation adjusted for the Japanese population (25). We also measured PG, hemoglobin A1c (HbA1c), B-type natriuretic peptide (BNP), serum total cholesterol, triglycerides (TG), high-density lipoprotein cholesterol (HDL-C), and insulin levels. Low-density lipoprotein cholesterol (LDL-C) levels were calculated using the Friedewald formula (26). Plasma BNP levels were measured using a specific immunoradiometric assay for human BNP (ARCHITECT BNP-JP^®^, ABBOTT JAPAN Co., Ltd., Tokyo, Japan).

### Body Composition Analysis

Body composition was analyzed using a bioelectrical impedance analysis device (InBody720/S10, Biospace Co., Ltd., Tokyo, Japan). This method is based on the principle that lean body mass contains higher water and electrolyte content than fat tissue; hence, these tissues can be distinguished by electrical impedance. Segmental body composition was estimated using a patented 8-point tactile electrode system. The device uses six frequencies (1, 5, 50, 250, 500, and 1000 kHz) and produces 30 impedance values for five body segments: the right and left upper extremities, trunk, and right and left lower extremities (27). A previous validation study demonstrated that both fat mass and lean body mass content measured using this device were highly correlated with measurements using dual-energy X-ray absorptiometry (28).

### Statistical Analysis

Statistical analyses were performed using SPSS version 23 (IBM Co., Ltd., Chicago, IL, USA). All values are expressed as mean ± SD. Pearson’s correlation coefficient was calculated to analyze the association of serum Zn/Cu ratio with physical, biochemical, and physiological data. Multiple regression analysis was performed to test independent correlations between serum Zn/Cu ratio and clinical data. Differences in clinical data between patients with and without type 2 diabetes were analyzed using the paired *t*-test. Furthermore, multivariate logistic regression analyses were performed to analyze the association between the quartile of serum Zn/Cu ratio and glycemic control (good: HbA1c < 7.0%; poor: HbA1c ≥ 7.0%) in patients with type 2 diabetes and to determine odds ratios and 95% confidence intervals (CI). *p* values < 0.05 were considered statistically significant.

## Results

### Characteristics of Subjects

A total of 355 subjects (151 men and 204 women) were included in this study. The mean age was 61.1 ± 17.6 years. The number of patients with type 2 diabetes was 149 (42.0%). The number of patients with dyslipidemia, hypertension, CKD, and liver diseases such as fatty liver were 117 (33.0%), 94 (26.5%), 87 (24.5%), and 35 (9.9%), respectively. Seventy two patients (20.3%) suffered from autoimmune diseases such as Hashimoto disease, Sjögren’s Syndrome, and rheumatoid arthritis. Thirty-three patients (9.3%) suffered from endocrine disorders such as adrenal deficiency and hyper/hypothyroidism. Eighteen patients (5.1%) had malignant diseases, such as lung cancer, prostate cancer, breast cancer, gastric cancer, and colon cancer. Eleven patients (3.1%) had neurodegenerative diseases, such as Parkinson disease and Alzheimer dementia. The number of patients who had a history of CVD was 29 (8.2%). Patient characteristics are summarized in Table 1. The prevalence of dyslipidemia (*p* < 0.001), hypertension (*p* < 0.001), and CVD (*p* < 0.001) was higher and the prevalence of endocrine disorders (*p* = 0.005) and autoimmune diseases (*p* < 0.001) was lower in patients with type 2 diabetes than in those without (by chi-square test).

**Table 1:** **Patient clinical characteristics**.

Demographics	
*n*	355
Age, years	61.1 (17.6)
Sex (male/female)	151/204
Height, cm	159.4 (9.4)
Weight, kg	61.6 (18.1)
BMI, kg/m^2^	24.1 (5.7)
**Blood data**	
Red blood cell, ×10^4^/μl	430 (55.3)
Hemoglobin, g/dl	13.1 (1.5)
Hematocrit, %	38.8 (5.2)
**Biochemical data**	
Albumin, g/dl	4.2 (0.5)
Blood urea nitrogen, mg/dl	15.1 (7.2)
Creatinine, mg/dl	0.8 (0.3)
eGFR, ml/min/1.73 m^2^	75.4 (26.9)
Total cholesterol, mg/dl	187.4 (39.9)
Triglycerides, mg/dl	135.1 (91.5)
HDL cholesterol, mg/dl	54.5 (15.8)
LDL cholesterol, mg/dl	105.5 (31.5)
Plasma glucose, mg/dl	127 (60.6)
HbA1c, %	6.8 (1.8)
Serum insulin, μU/ml (*n* = 42)	11.6 (7.7)
Plasma BNP, pg/ml (*n* = 80)	53.9 (71.1)
Serum Zn, μg/dl	72.6 (15.4)
Serum Cu, μg/dl	110.8 (26.4)
Serum Zn/Cu ratio	0.69 (0.21)
**Body composition** (***n* = 119**)	
Skeletal muscle mass (whole body), kg	26.1 (6.7)
Right upper extremity muscle mass, kg	2.5 (0.8)
Left upper extremity muscle mass, kg	2.5 (0.8)
Right lower extremity muscle mass, kg	7.3 (1.9)
Left lower extremity muscle mass, kg	7.2 (1.9)
Body fat mass, kg	22.8 (12.5)
Body fat percentage, %	30.8 (10.7)

*Values are expressed as means (SD) except for number of subjects and sex*.

*BMI, body mass index; eGFR, estimated glomerular filtration rate; HDL, high-density lipoprotein; LDL, low-density lipoprotein; HbA1c, hemoglobin A1c; BNP, B-type natriuretic peptide*.

### Associations between Serum Zn/Cu Ratio and Clinical Parameters in All Subjects

Serum Zn/Cu ratio was inversely correlated with age and plasma BNP levels, whereas it was positively correlated with height, weight, serum Alb levels, and eGFR. Serum Zn/Cu ratio was also positively correlated with red blood cell count, Hb, Ht, and skeletal muscle mass (Table 2).

**Table 2:** **Correlations between serum Zn/Cu ratio and clinical parameters in all subjects**.

	Correlation coefficient	*p*-Value
**Demographics**		
Age	−0.284	<0.001
Height	0.219	<0.001
Weight	0.152	0.005
BMI	0.07	0.2
**Blood data**		
Red blood cell	0.192	0.003
Hemoglobin	0.302	<0.001
Hematocrit	0.197	0.003
**Biochemical data**		
Albumin	0.357	<0.001
eGFR	0.144	0.008
Total cholesterol	−0.046	0.44
Triglycerides	0.108	0.062
HDL cholesterol	−0.063	0.3
LDL cholesterol	−0.039	0.5
Plasma glucose	−0.047	0.42
HbA1c	−0.085	0.16
Serum insulin	0.123	0.44
Plasma BNP	−0.383	<0.001
**Body composition**		
Skeletal muscle mass (whole body)	0.189	0.039
Right upper extremity muscle mass	0.183	0.046
Left upper extremity muscle mass	0.193	0.036
Right lower extremity muscle mass	0.214	0.019
Left lower extremity muscle mass	0.224	0.014
Body fat mass	−0.064	0.49
Body fat percentage	−0.152	0.099

*eGFR, estimated glomerular filtration rate; HDL, high-density lipoprotein; LDL, low-density lipoprotein; HbA1c, hemoglobin A1c; BNP, B-type natriuretic peptide*.

Positive associations of serum Zn/Cu ratio with serum Alb levels and Hb remained after adjustment for age, sex, and BMI (β = 0.335, *p* < 0.001 and β = 0.237, *p* = 0.002, respectively). Plasma BNP levels were also negatively associated with serum Zn/Cu ratio (β = −0.258, *p* = 0.032). In addition, eGFR was positively associated with serum Zn/Cu ratio after adjustment for BMI (β = 0.137, *p* = 0.014). However, independent associations between serum Zn/Cu ratio and other biochemical parameters were not detected.

To investigate differences in clinical parameters between patients with and without type 2 diabetes, patients with cancers, autoimmune diseases, endocrine disorders, and liver dysfunction, which were known to affect serum levels of Zn and Cu, were excluded from the analysis. Height, weight, BMI, red blood cell count, and Hb were significantly higher in patients with type 2 diabetes than those without type 2 diabetes. Serum levels of HDL-C were significantly lower in patients with type 2 diabetes than those without type 2 diabetes (Table 3).

**Table 3:** **Comparison of clinical data between patients with and without type 2 diabetes**.

	Type 2 diabetic patients (*n* = 113)	Non-diabetic subjects (*n* = 110)	*p*-Value
Age, years	64.4 (14.7)	60.7 (20.4)	0.12
Duration of diabetes, years	10.5 (10)	–	–
Sex (male/female)	64/49	36/74	<0.001
Height, cm	161 (8.8)	157.5 (10.2)	0.009
Weight, kg	68.8 (17.9)	57.9 (17.7)	<0.001
BMI, kg/m^2^	26.4 (5.6)	23.2 (5.6)	<0.001

Red blood cell, ×10^4^/μl	445.6 (61.2)	423.5 (50.2)	0.02
Hemoglobin, g/dl	13.7 (1.7)	13 (1.4)	0.013
Hematocrit, %	40.1 (5.8)	38.2 (4.8)	0.2

Albumin, g/dl	4.1 (0.5)	4.3 (0.5)	0.028
eGFR, ml/min/1.73 m^2^	71.2 (25.1)	79 (32.9)	0.054
Total cholesterol, mg/dl	184.9 (37.4)	192.7 (41.5)	0.18
Triglycerides, mg/dl	138.6 (88.7)	130.6 (85.2)	0.53
HDL cholesterol, mg/dl	51.5 (16.3)	58.1 (15.2)	0.007
LDL cholesterol, mg/dl	104.1 (30.8)	108.3 (31.5)	0.35
Plasma glucose, mg/dl	151.5 (62.2)	99.5 (21)	<0.001
HbA1c, %	7.7 (1.9)	5.7 (0.4)	<0.001
Serum insulin, μU/ml	10.9 (7.1)	13.1 (10)	0.56
Plasma BNP, pg/ml	55.5 (97.3)	62.5 (59.6)	0.76
Serum Zn, μg/dl	75.8 (13.7)	72.7 (18.7)	0.15
Serum Cu, μg/dl	109.9 (21)	109.4 (23)	0.42
Serum Zn/Cu ratio	0.72 (0.19)	0.69 (0.23)	0.87

Skeletal muscle mass (whole body), kg	26.9 (6.4)	25.3 (7.2)	0.28
Right upper extremity muscle mass, kg	2.7 (0.8)	2.4 (0.9)	0.13
Left upper extremity muscle mass, kg	2.6 (0.8)	2.4 (0.9)	0.16
Right lower extremity muscle mass, kg	7.6 (1.9)	7.2 (2.1)	0.36
Left lower extremity muscle mass, kg	7.5 (1.9)	7.1 (1.9)	0.33
Body fat mass, kg	23.9 (13.2)	23 (11.8)	0.76
Body fat percentage, %	31.2 (10.1)	31.9 (10.2)	0.75

*Values are expressed as means (SD) except for sex*.

*BMI, body mass index; eGFR, estimated glomerular filtration rate; HDL, high-density lipoprotein; LDL, low-density lipoprotein; HbA1c, hemoglobin A1c; BNP, B-type natriuretic peptide*.

**Figure 1:**
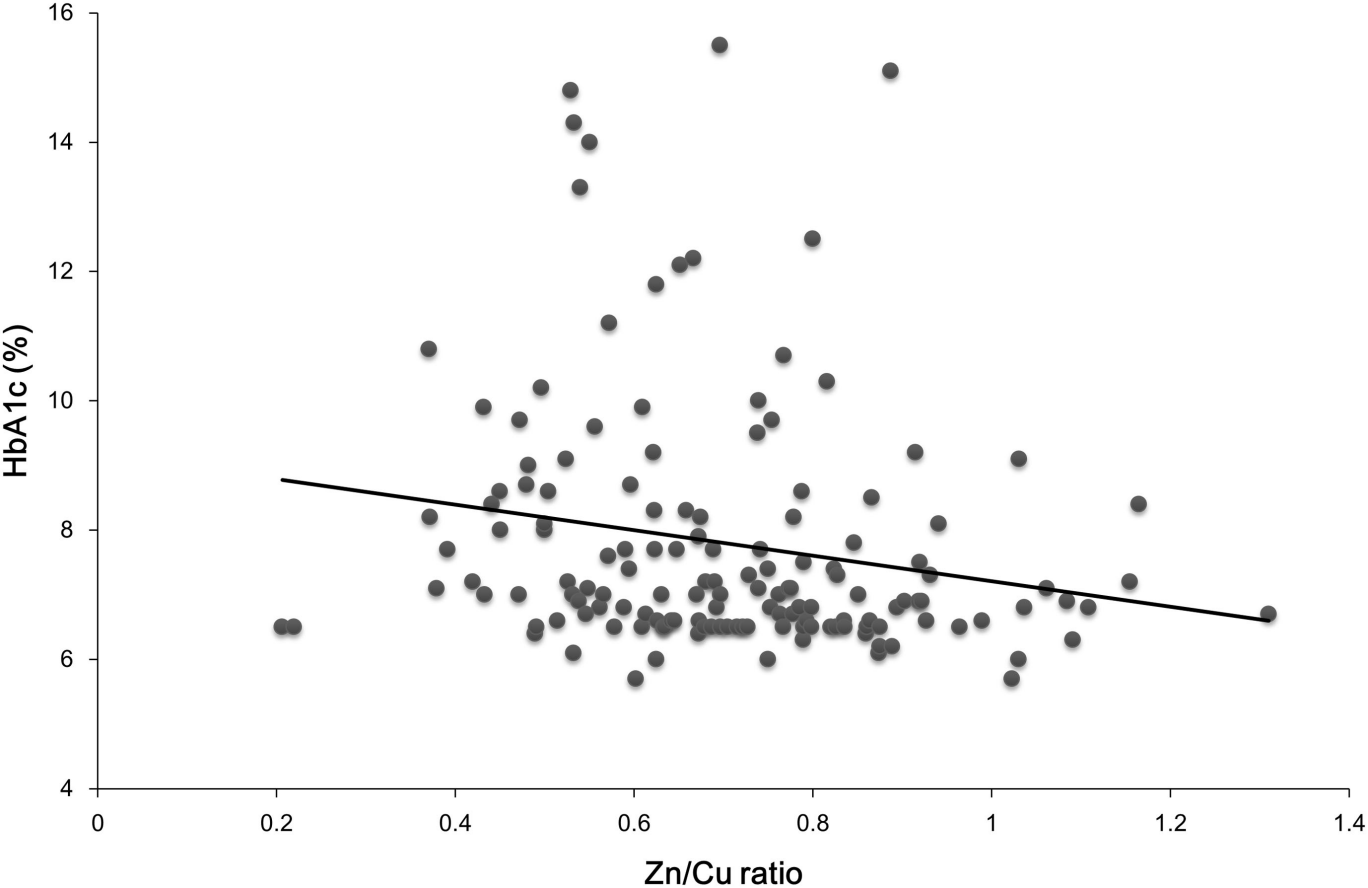
**An inverse correlation between serum Zn/Cu ratio and plasma HbA1c levels in patients with type 2 diabetes**.

The prevalence of dyslipidemia was higher in patients with type 2 diabetes than that in patients without diabetes (*p* < 0.001). However, there were no differences in the prevalence of other diseases, such as hypertension (*p* = 0.065) and CKD (*p* = 0.23), between patients with and without type 2 diabetes. In patients with type 2 diabetes, serum Zn/Cu ratio was positively correlated with height (*r* = 0.178, *p* = 0.031), serum levels of Alb (*r* = 0.46, *p* < 0.001), Hb (*r* = 0.41, *p* < 0.001), and Ht (*r* = 0.277, *p* = 0.016) and negatively correlated with age (*r* = −0.223, *p* = 0.006), plasma HbA1c levels (*r* = −0.193, *p* = 0.019) (Figure 1), and plasma BNP levels (*r* = −0.392, *p* = 0.022). Positive associations of serum Zn/Cu ratio with serum Alb levels and Hb were observed after adjustment for age, sex, and BMI (β = 0.494, *p* < 0.001 and β = 0.476, *p* < 0.001, respectively), and serum Zn/Cu ratio remained negatively associated with plasma HbA1c levels after the same adjustments (β = −0.239, *p* = 0.003). However, significant associations between serum Zn/Cu ratio and other biochemical data disappeared following statistical adjustments.

The multivariate logistic regression analysis revealed that the highest quartile of serum Zn/Cu ratio was significantly associated with a decreased risk of poor glycemic control (odds ratio = 0.382; 95% CI, 0.165–0.884; *p* = 0.025) in patients with type 2 diabetes (Table 4).

**Table 4:** **Logistic regression analysis of glycemic control in patients with type 2 diabetes**.

	Adjusted odds ratio	95% CI	p-Value
Age	1.003	0.980–1.021	0.78
Sex			
Male	0.844	0.440–1.618	0.61
Female	(reference)		
BMI	1.045	0.982–1.111	0.16
**Serum Zn/Cu ratio**			
<0.5461	(reference)		
0.5462–0.6721	0.638	0.298–1.364	0.25
0.6722–0.8	0.604	0.281–1.295	0.19
>0.8	0.382	0.165–0.884	0.025

*The odds ratio of good glycemic control (HbA1c < 7.0%) to poor glycemic control (HbA1c ≧ 7.0%) was calculated*.

*HbA1c, hemoglobin A1c; CI, confidence interval; BMI, body mass index*.

## Discussion

To the best of our knowledge, this is to be the first study to demonstrate the associations of serum Zn/Cu ratio with renal function, plasma BNP levels, and glycemic control in a relatively large population.

A positive correlation between serum Zn/Cu ratio and serum Alb and Hb levels is expected. Plasma Zn concentrations are reduced by hypoalbuminemia, as zinc is bound to Alb in the circulation, and Zn deficiency is a known cause of anemia (29). Although Cu deficiency is a potential cause of hematologic abnormalities (30), no significant associations between serum Cu levels and blood cell count were observed in this study.

An imbalance between Zn and Cu levels leads to increased oxidative damage in humans, contributing to the pathogenesis of diabetes and diabetic complications (1, 2, 31). The dysregulation of matrix metalloproteinases which include a Zn ion-binding site are associated with the progression of diabetic complications *via* the interaction with advanced glycation end products in patients with type 2 diabetes, although the relationship between matrix metalloproteinases and serum Zn levels were not investigated in the present study (12). Furthermore, these trace elements are necessary for the activity of mitochondrial antioxidant enzymes, such as Cu/Zn superoxide dismutase (SOD), that protect the cell from reactive oxygen species toxicity (32). Both Zn and Cu are essential for metabolism; however, an imbalance in Zn/Cu (or Cu/Zn) ratio may be a better indicator of metabolic disturbance than Zn or Cu status alone. Indeed, previous studies have demonstrated the utility of Cu/Zn ratio as a biomarker for vascular complications in type 2 diabetes (33) and a predictor of mortality in elderly individuals (34).

Renal dysfunction has been shown to be induced by Cu toxicity in both rats (35) and humans (36). On the other hand, Zn may contribute to the preservation of renal function. Kurihara et al. reported that Zn deficiency decreased renal blood flow and increased renal vascular resistance, which may be attributable to decreased nitric oxide activity due to the presence of increased concentrations of superoxide anions through low SOD activity in the kidneys of rats (37). Yanagisawa et al. suggested that Zn deficiency aggravates tubulointerstitial nephropathy in rats due to an increase in the action of angiotensin II and endothelin (38). Recently, Sun et al. demonstrated that renal function was improved by Zn supplementation in diabetic mice (39). Zn supplementation has also been shown to reduce Alb excretion in patients with diabetic nephropathy (40, 41). The role of Zn is of importance in preserving renal function. However, there is a lack of evidence regarding the association between Zn and/or Cu and renal function in humans. It is notable that the results of this study indicate the potential utility of serum Zn/Cu ratio as a biomarker for renal function.

The negative association observed between serum Zn/Cu ratio and plasma BNP levels in this study may be mediated by insulin sensitivity. An inverse association between natriuretic peptides and insulin resistance has been reported by previous studies (42–44). BNP stimulates lipolysis (45) and promotes muscle mitochondrial biogenesis and fat oxidation through upregulation of peroxisome proliferator-activated receptor-γ coactivator-1α (PGC-1α) (46) and increases adiponectin secretion (47), thereby improving insulin resistance. In addition to previous reports of the association of an imbalance in Zn and Cu levels with increased oxidative stress and inflammation (34, 48), which impair insulin secretion and action (1), Zn deficiency may lower SOD1 activity in pancreatic islets, which has been shown to increase insulin resistance (49, 50). Zn has also been shown to be associated with insulin secretion *via* metallothionein synthesis (51) and the zinc transporter ZnT8 (15). Recently, Zn has been observed to promote insulin secretion independently on metallothionein synthesis (52). The inverse association between serum Zn/Cu ratio and plasma BNP levels observed in this study after adjustment for age, sex, and BMI indicates that a higher Zn/Cu ratio may be associated with improved insulin sensitivity. However, we were unable to detect a significant association between serum Zn/Cu ratio and insulin levels. This inconsistency may be because of the following study limitations: the small sample size (*n* = 42) for investigating the association between serum Zn/Cu and insulin and confounding effects of oral hypoglycemic agents and diet. We are unable to confidently explain the negative association observed between serum Zn/Cu ratio and BNP in this study. Thus, further studies are required to elucidate the mechanisms underlying the association between serum Zn/Cu ratio and BNP.

Several studies have demonstrated decreased serum/plasma Zn levels in patients with type 2 diabetes compared with healthy individuals (53–55); on the contrary, serum Zn levels were not lower in patients with type 2 diabetes in this study. As mentioned above, serum Zn levels were found to be strongly affected by nutritional status and Hb levels; thus, no significant difference in serum Zn levels between patients with and without type 2 diabetes was observed.

Low HDL-C levels in patients with type 2 diabetes may have been observed as serum HDL-C levels have been shown to decrease with impaired function in type 2 diabetes (56). Patients with type 2 diabetes were more obese than those without type 2 diabetes in this study. Although no significant differences in body composition between patients with and without type 2 diabetes were found, obesity may be attributable to lower HDL-C levels in patients with type 2 diabetes.

The observed associations of serum Zn/Cu ratio with lower HbA1c levels and reduced risk of poor glycemic control (HbA1c ≥ 7.0%) in a population of patients with type 2 diabetes are noteworthy findings of this study. Although excess of intracellular Zn can trigger oxidative stress from mitochondria and lead to neuronal degeneration (57), Zn has previously been shown to have beneficial effects on glycemic control as well as aging, immunity, and oxidative stress (58), whereas serum Cu is associated with higher HbA1c levels (31, 59). Experimental studies have shown that the amino acids residues involved in Cu coordination complexes have a key role in the formation of human islet amyloid peptide aggregation, which alters the autophagy pathway in pancreatic β-cells and leads to the development of diabetes (19, 20). The actions of Zn and Cu in glucose metabolism appear to be antagonistic; hence, the balance between Zn and Cu ion concentrations is important. In this study, higher Zn levels were not associated with good glycemic control (odds ratio = 0.991; 95% CI, 0.968–1.014; *p* = 0.436), whereas serum Cu levels were associated with glycemic control (odds ratio = 1.024; 95% CI, 1.007–1.041, *p* = 0.006). However, the high serum Zn/Cu ratio was associated with good glycemic control in patients with type 2 diabetes. This result suggests that not only Zn and Cu status alone but also Zn/Cu ratio should be considered when evaluating the relationship between these trace elements in patients with diabetes.

This study had several limitations. First, a causal relationship between serum Zn/Cu ratio and renal function and/or glycemic control could not be evaluated because of the retrospective and observational nature of this study. Second, we were unable to evaluate the utility of other biomarkers in assessing Zn and Cu status, such as urinary and other tissue concentrations of Zn and Cu. Zn and Cu concentrations are known to be affected by other factors, such as inflammation, and fasting or postprandial states (60, 61). However, blood Zn and Cu levels are currently considered to be useful and reliable biomarkers (60, 61). Third, we are unable to generalize the results of this study to healthy individuals or other populations. The subjects included in this study had a high prevalence of comorbid diseases, such as hypertension, dyslipidemia, fatty liver, autoimmune disease, CKD, and CVD, besides type 2 diabetes. Neurodegenerative disorders could also undergo changes in serum Zn and Cu levels. Although we excluded subjects with malnutrition and anemia, comorbidities and medications may have affected the observed associations of Zn and Cu status with other parameters. We should also perform a further investigation in healthy controls. Fourth, other confounding factors, such as dietary intake, smoking and drinking habits, and physical activity, should also be considered. Fifth, we were unable to provide a value for the most desirable balance between Zn and Cu from the results of this study. Despite these limitations, we were able to demonstrate significant associations of serum Zn/Cu ratio with renal function, glycemic control, and a number of metabolic parameters, providing new insights in the management of type 2 diabetes.

## Conclusion

The findings of this study demonstrate that serum Zn/Cu ratio is significantly associated with renal function in all subjects and glycemic control in patients with type 2 diabetes. Serum Zn/Cu ratio was negatively associated with plasma BNP levels. Imbalance between Zn and Cu levels induces oxidative stress and insulin resistance, which may lead to progression of diabetes and diabetic complications. Zn/Cu ratio, in addition to individual levels of each trace element status alone, appears to have an important effect on metabolism, indicating that these trace elements may play a key role in the pathogenesis of metabolic diseases. We, clinicians should note that serum Zn/Cu ratio could be a better indicator for human metabolism as compared with Zn or Cu status alone.

## Author Contributions

HH performed the study, conducted the data analyses, and drafted and revised the manuscript. YK contributed to the data collection and analyses. HY critically reviewed the manuscript and the scientific interpretations of study results. All the authors read and approved the final manuscript.

## Conflict of Interest Statement

The authors declare that the research was conducted in the absence of any commercial or financial relationships that could be construed as a potential conflict of interest.
